# Sudden Death from the Long-continued Use of Carbonate of Soda

**Published:** 1851-01

**Authors:** 


*Sudden Death from the long-continued use of Carbonate of Soda.*—Dr. Tunstall, of the Bath Dispensary, reports in the London Medical Times, November 30, 1850, a case of very sudden death, traceable to the abuse of alkalies. The subject, a tall muscular man, 53 years old, of temperate habits, dropped down dead in the streets of Bath. About eighteen years since he first began to complain of severe dyspepsia, for which he was advised to take a small quantity of the bicarbonate of soda; when necessary, this quantity he had gradually increased until he took half a hundred weight yearly, or rather more than two ounces daily, for sixteen years. During this period he had two severe and long continued fits, the last five years since, when he remained cold, insensible, and passive, for nearly thirty minutes. He had been travelling for twenty-four hours previously to his death by a stage wagon, had alighted for the purpose of inquiring his way, and, while in the act of speaking to his daughter, fell dead. He had dined five hours before, and had drunk half a pint only of beer; had taken several doses of soda on the road, more than a pound of which was found in his pocket when the body was searched.

A post-mortem examination was obtained only of the abdominal cavity. The following is the account of it:—Externally the body was blanched, and upon making an incision little cellular or adipose tissue was met with; the muscles were pallid, resembling veal after bleeding; upon opening the abdomen the stomach was discovered enlarged to twice its natural size, occupying the whole of the superior third of the abdominal cavity; its peritoneal surface was covered with reticulated blood vessels, and a bright brick-red blush, commencing with the cardiac extremity, extended over one-third of its surface. Upon opening it the whole mucous membrane was found disintegrated and pulpy. Numerous ulcerations, varying in size from a pea to a large bean, were scattered throughout, so that its first appearance was like that of a polished tortoise shell. During its removal from the body, which was carefully done by Mr. Goldstone, its coats gave away to the extent of three inches, leaving a rough edge; the stomach contained half a pint of a strong acid fluid, with portions of meat in a partially digested condition; the small intestines presented similar appearances to those found in the stomach, and there was little or no fat in the omentum.

The liver was contracted in size and consolidated, as was the spleen.

Dr. Tunstall was inclined, he says, at the first view, to believe that it had arisen from nervous apoplexy, but this idea was negatived by the fact, that up to the moment of his death he had been in conversation with his daughters, and that death was instantaneous and complete; there was no evidence of a rupture of the heart or great vessels, for I counted three distinct pulsations under my hand; so that I think we may safely affirm that the fatal attack was one of syncope, produced by the attenuation of the circulating mass by the long continued use of the alkali.

That a great analogy exists between the effects of long-continued alkaline remedies and scurvy is well pointed out in Pereira's "Elements of Materia Medica," where, speaking of liquor potassæ, he says, "it appears in the highest degree probable, that scurvy, and the effects caused by the long-continued employment of the alkalies, are analogous conditions of system.

Carbonate of soda has, within the last few years, become almost an established article of diet of daily consumption in our tea, and for the purpose of neutralizing the acidity of malt liquors; so that, apart from the curiosity excited by the present case, I am anxious that its relation should have a practical importance; that it may be taken with supposed impunity, in large doses, for a period of sixteen years, the present case testifies. We have a laborious artisan, never complaining of severe illness, taking what he supposed to be an innocent remedy for dyspepsia, and who, no doubt, had he complained of illness, would not have thought it of sufficient importance to mention, as his daughter did at the inquest, until Mr. Goldstone suggested the question to the Coroner.

